# Medical Botany, &c.

**Published:** 1847-08

**Authors:** 


ARTICLE XII.

*Medical Botany, or Descriptions of the more important Plants used in Medicine, with their History, Properties, and Mode of Administration.* By R. EAGLESFIELD GRIFFITH, M.D., Member of the American Philosophical Society; of the Academy of Natural Sciences of Philadelphia, etc., etc.; with upwards of three hundred illustrations. Philadelphia: Lea & Blanchard. 1847. 8vo., pp. 704. (From the Publishers, and for sale by Joseph Keen, Jr., Chicago.)

This is a work which will be greeted with much pleasure by all who may obtain possession of it. It is by far the most comprehensive and complete work upon the subject which has issued from the American press. It indeed fills a great vacancy in the medical literature of the country. This subject—Medical Botany—has been but too much neglected in this country, both by students and practitioners; and we think the cause has been, in a measure, the want of a good text-book or work of reference upon the subject. In our works upon materia medica, no reference is had, in their arrangement, to botanic classification, and hence they afford no aid to this important study, further than the *isolated* description of plants used in medicine. The book before us supplies all the wants of the professions in this regard, and we recommend it to them as all they could desire in a work upon this subject. The introdution is a brief comprehensive essay on the structure and composition of plants. "A Glossary of Terms," and a "Conspectus of the natural order of plants containing Medical Substances," materially add to the value of the work. An "Index of the common and foreign names of species and of vegetable products," and another of "Orders, Genera, and Species, with their Synonymes," afford great facilities for reference. The illustrations are copious, and excellent in design and execution. For mechanical execution, the name of the well-known house who publish the work will afford sufficient guarantee.

J. V. Z. B.

